# Management of Vesicoureteral Reflux: What Have We Learned Over the Last 20 Years?

**DOI:** 10.3389/fped.2021.650326

**Published:** 2021-03-31

**Authors:** Göran Läckgren, Christopher S. Cooper, Tryggve Neveus, Andrew J. Kirsch

**Affiliations:** ^1^Section of Urology, Department of Pediatric Surgery, University Children's Hospital, Uppsala, Sweden; ^2^Department of Urology, University of Iowa, Iowa City, IA, United States; ^3^Department of Women's and Children's Health, Uppsala University, Uppsala, Sweden; ^4^Pediatric Urology, Children's Healthcare of Atlanta and Emory University School of Medicine, Atlanta, GA, United States

**Keywords:** antibiotic, bladder/bowel dysfunction, endoscopic injection, NASHA/Dx, vesicoureteral reflux, ureteral reimplantation, urinary tract infection, voiding cystourethrogram

## Abstract

Vesicoureteral reflux (VUR) is associated with increased risks of urinary tract infection, renal scarring and reflux nephropathy. We review advancements over the last two decades in our understanding and management of VUR. Over time, the condition may resolve spontaneously but it can persist for many years and bladder/bowel dysfunction is often involved. Some factors that increase the likelihood of persistence (e.g., high grade) also increase the risk of renal scarring. Voiding cystourethrography (VCUG) is generally considered the definitive method for diagnosing VUR, and helpful in determining the need for treatment. However, this procedure causes distress and radiation exposure. Therefore, strategies to reduce clinicians' reliance upon VCUG (e.g., after a VUR treatment procedure) have been developed. There are several options for managing patients with VUR. Observation is suitable only for patients at low risk of renal injury. Antibiotic prophylaxis can reduce the incidence of UTIs, but drawbacks such as antibiotic resistance and incomplete adherence mean that this option is not viable for long-term use. Long-term studies of endoscopic injection have helped us understand factors influencing use and the effectiveness of this procedure. Ureteral reimplantation is still performed commonly, and robot-assisted laparoscopic methods are gaining popularity. Over the last 20 years, there has been a shift toward more conservative management of VUR with an individualized, risk-based approach. For continued treatment improvement, better identification of children at risk of renal scarring, robust evidence regarding the available interventions, and an improved VUR grading system are needed.

## Introduction

Vesicoureteral reflux (VUR) is associated with increased risks of urinary tract infection and renal scarring or reflux nephropathy (1). Reflux nephropathy in children with VUR may be attributable to scars from upper urinary tract infection (UTI) as well as congenital renal dysplasia (1). The severity of VUR is described by a grading system according to the findings of a voiding cystourethrogram (VCUG), with grades ranging from I (mild) to V (severe). In most cases, VUR does not directly cause any symptoms; it is diagnosed either antenatally in children with hydronephrosis, or later following the occurrence of symptomatic UTIs (2, 3). Diagnosing the condition can be challenging due to the lack of direct symptoms and, in neonates and young infants, this is compounded by the non-specific manner with which UTIs present. Estimated prevalence rates for VUR range between 0.4 and 1.8% (4, 5).

Numerous studies have examined the links between VUR, UTIs, pyelonephritis, renal scarring and impaired renal function. In a study of 115 infants with grade III–V reflux, single-kidney glomerular filtration rate (GFR) was below 40% of the individual's total expected value in 71% of the patients, and a deterioration in renal status was observed in 18% of the patients (6). Recurrent febrile UTIs (fUTIs), bilateral renal abnormalities and reduced total GFR were identified as risk factors for renal deterioration. Swerkersson et al. evaluated VUR and renal scarring in children aged <2 years presenting with UTI (7). VUR and renal scarring were each present in 26% of the study participants, and the rate of renal abnormality increased significantly with increasing grade of VUR. A later study by the same group assessed changes over time in children aged <2 years presenting with UTI who were found to have renal scarring (8). Over a follow-up time of at least 2 years, 19% of the children exhibited renal deterioration. Grade III–V VUR and recurrent UTI were identified as risk factors for deterioration. Hidas et al. developed an instrument for predicting the risk of breakthrough UTI in children with VUR (9). VUR grade, gender, circumcision status, presence of bladder/bowel dysfunction (BBD) and cause of presentation of VUR enabled stratification of children into different risk groups. When the instrument was applied to a validation cohort, the predicted 2-year incidence of breakthrough UTI was 19.5%, compared with an actual rate of 21% (9). Arlen et al. similarly developed a tool for calculating the risk of a breakthrough fUTI in children with VUR based on risk factors for UTIs [including age, gender, VUR grade, reflux at low bladder volume, bladder/bowel dysfunction (BBD) and UTI history] (10). In a cohort of 255 children, the calculator was shown to have 76% accuracy. A study by Keren et al. investigated risk factors for recurrent UTI and renal scarring in children aged 2–71 months who had experienced one or two febrile or symptomatic UTIs (11). VUR, BBD and renal scarring were all associated with increased likelihood of recurrent UTIs. In males, circumcisional status may also be an important risk factor for UTI. One review reported that circumcision is associated with an 87% reduction in the incidence of UTI among boys with high-grade VUR (12).

In a Turkish study of 156 children aged 0–16 years with UTIs, increasing grade of VUR was associated with increasing rates of renal scarring (13). A longitudinal study with median follow-up of 5.6 years was conducted to investigate the association between renal scarring and adverse renal outcomes in children with a diagnosis of UTI or VUR (14). Patients with, vs. without, renal scarring showed significantly increased risk of developing proteinuria (5.1 vs. 1.6%, *p* = 0.005) and kidney disease (2.0 vs. 0.0%, *p* = 0.005). The available data support intervention in patients with VUR to reduce the risks of pyelonephritis and renal scarring, which can have permanent consequences.

VUR has long been known to resolve spontaneously over time. However, a decision to wait for this to occur rather than treating or curing the condition should only be taken in the absence of repeat fUTIs that could cause renal scarring. In 1998, Wennerström et al. reported that grade III–V reflux resolved spontaneously (to grade 0–I) in 73% of cases over a follow-up period of 10 years (15). Early investigations also showed that older age, high-grade VUR and female gender were associated with a lower likelihood of spontaneous VUR resolution (15, 16). Later studies identified high-grade VUR, renal abnormalities, prenatal hydronephrosis, bladder dysfunction, low bladder filling volume at reflux onset, breakthrough UTI and older age upon diagnosis of VUR as independent predictors of a lower likelihood of spontaneous resolution (17–21). Evidence suggests that effective treatment of BBD can increase the chance of spontaneous resolution of VUR (22). Kirsch et al. performed multivariate analysis on outcomes from 229 patients diagnosed with VUR before the age of 2 years, and reported that patients with the following had significantly longer time to spontaneous resolution: grade IV–V VUR, duplicated ureters or periureteral diverticula, occurrence of reflux during bladder filling, and female gender (23). The occurrence of reflux early during bladder filling has been associated with low spontaneous resolution rates and increased risk of fUTI, independent of the grade of VUR (18, 23, 24).

VUR and BBD are closely related and around half of patients with VUR also have BBD (25). Among patients with VUR, additional presence of BBD approximately doubles the risk of UTIs (11, 25, 26). As mentioned above, co-existent BBD may also reduce the likelihood of spontaneous resolution of VUR, and BBD has been associated with reduced success in patients undergoing endoscopic injection for VUR (17, 27). On the other hand, intervention for VUR can lead to the improvement or cure of BBD, indicating a degree of interdependence between the two conditions (28–30). Treatment of BBD as well as VUR in patients with both conditions appears to be advisable (22, 27).

In females, VUR is associated with increased risk of pregnancy-related complications such as pre-eclampsia and UTI (31, 32). This is mainly attributable to the presence of renal scarring, supporting the notion that preventing renal damage should be a key goal of VUR management. However, UTI prevention may also be important since the risk of fetal complications is elevated among women with frequent UTIs (32).

There are four main options for managing patients with VUR: observation, antibiotic prophylaxis, endoscopic injection and ureteral reimplantation (33–35).

## Diagnosis and Assessment

The key aims of assessment are to determine how and when the patient should undergo treatment. VCUG has been described as the only definitive method of diagnosing VUR and defining its severity (36–38). However, inter-rater variability is common with this assessment. Some studies have reported favorable intraclass correlation coefficient (ICC) values between 0.8 and 0.9, but lower levels of inter-rater agreement (50–60%) have also been reported (39–42). To improve the reliability of results, a standardized protocol should be adhered to when performing this assessment (37, 43). Specifications such as choice of contrast, method for infusing contrast, timing and quality of spot images, and documentation of bladder volume at onset of VUR ensure consistency (37). Also, more than one cycle of filling and voiding may be needed to avoid the possibility of underdiagnosing VUR (44). Optimal VCUG methodology is not always followed, and this represents an opportunity to improve routine clinical practice (43).

Perceived importance of the available assessments of VUR patients has changed significantly over the last 20 years. Historically, the grade of VUR was often the only determinant of treatment decisions. The VCUG procedure causes considerable distress and exposes patients to radiation. Strategies to reduce clinicians' reliance upon VCUG have therefore been developed (45). As well as the grade of VUR, treatment decisions are routinely based on age, gender, UTI occurrence and the presence of renal scarring. Determination if the patient has BBD is particularly important. Reproducible, validated methods for diagnosing BBD are limited and an 18-item questionnaire developed in 2019 was shown to enable reliable diagnosis and subcategory classification (46). VCUG assessment is considered necessary in patients with recurrent fUTIs (38, 47, 48). For children aged 2–24 months presenting with their first fUTI, routine VCUG is supported by the American Academy of Pediatrics only if there is an abnormality on a renal and bladder ultrasound scan (36, 49). Assessments other than VCUG that can help determine appropriate therapies include the frequency of UTIs or fUTIs, renal function tests [e.g., dimercaptosuccinic acid (DMSA) or mercaptoacetyltriglycine (MAG-3) scanning], ultrasound scanning, and assessment of bowel and bladder function (49–54).

**Figure 1:**
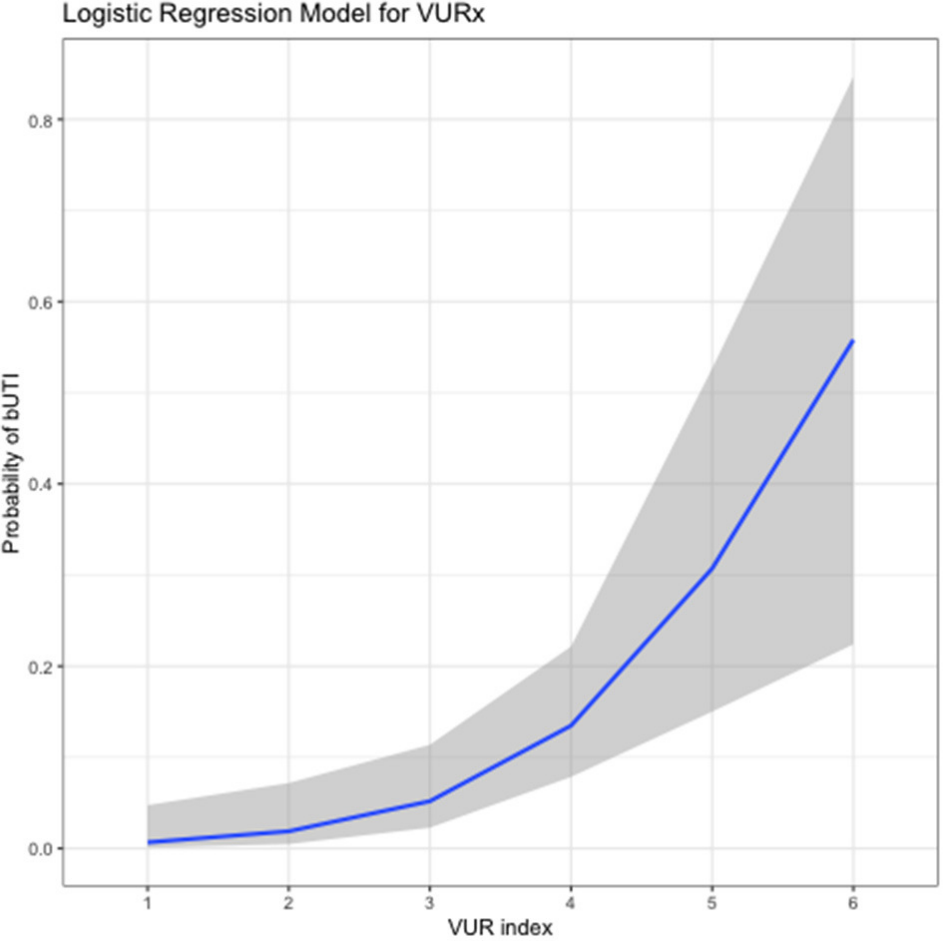
Relationship between vesicoureteral reflux index (VURx) score and subsequent occurrence of breakthrough urinary tract infections (bUTIs) (63). The graph is based on data from a cohort of 139 patients, mean age at VUR diagnosis 5.45 years (standard deviation, 4.7 years), followed for a mean of 32.1 months (standard deviation, 24.5 months) after diagnosis. Reproduced with permission purchased from the Copyright Clearance Center.

A future shift toward assessing the severity of VUR in patients with methods that are more objective and easily measurable than the current grading system is recommended by the authors. The distal ureteral diameter ratio (UDR; diameter of distal ureter normalized to the L1–L3 vertebral body distance; measurable using VCUG images) has been shown to be predictive of spontaneous resolution of VUR and risk of breakthrough fUTI (55–58). Each unit increase of UDR of 0.1 is associated with a significant increase in the probability of VUR persistence (55, 56, 59). A significant decrease in inter-grader variability has been reported with the UDR assessment compared with VUR grading, with ICC values of 0.95 and 0.87, respectively (59). Knowledge of the factors involved in spontaneous resolution of VUR prompted the development of computational prediction methods (60). Subsequently, a VUR index was proposed, where a patient's clinical characteristics (e.g., gender, grade of VUR, timing of VUR) are used to predict the likelihood of spontaneous resolution (23). Reliability of this index was initially shown in a cohort of VUR patients aged <2 years (*n* = 229), and then validated in a second cohort of patients aged <2 years (*n* = 369) as well as in 271 patients aged >2 years (23, 61, 62). The VUR index score has also been shown to correlate with the risk of developing UTIs (Figure 1) (23, 62, 63). Importantly, both UDR and VUR index appear superior to international VUR grading in predicting either spontaneous resolution of VUR or risk of a breakthrough UTI in children aged <2 years at diagnosis (55, 61, 63). Contrast-enhanced voiding urosonography (ceVUS) could potentially be used as a replacement for VCUG. This method enables determination of the presence and grade of VUR in a similar manner to VCUG, without exposing the patient to ionizing radiation. Available evidence suggests that ceVUS may provide acceptable diagnostic accuracy (64–66). In one study using VCUG results as the reference point, ceVUS was shown to provide sensitivity of 92% and specificity of 98% (65). The concordance rate between the two methods in determining the grade of VUR was 82%. Further data are needed to establish the suitability of ceVUS for use in routine clinical practice.

## Treatment Approaches

The principal aim of VUR management is to reduce kidney infections and renal scarring. In addition, clinicians should aim to prevent UTIs and minimize long-term assessment and treatment procedures. Management may be non-surgical (e.g., urotherapy, antibiotic therapy), minimally invasive (endoscopic injection) or surgical (ureteral reimplantation), and these approaches are detailed below.

### Observation

The selection of “observation” may be perceived as favorable due to the avoidance of medical intervention. However, regular follow-up visits to the clinic are required to enable adequate monitoring of the patient's status. In addition, parents must always be vigilant to ensure that all UTIs are reported and managed. Antibiotic therapy should be administered promptly to treat fUTIs, while frequent occurrence of UTIs is an indication for a different management strategy (1, 22, 67). Observation is only considered suitable for patients with a relatively low risk of renal injury (i.e., males with low-grade VUR) (68–70).

### Antibiotic Prophylaxis

Antibiotic prophylaxis has been reported to be effective in preventing UTIs. In the randomized intervention for children with vesicoureteral reflux (RIVUR) trial, the risk of recurrent infection was reduced by 50% vs. placebo among VUR patients with one or two prior UTIs (71). Similarly, in the randomized Swedish Reflux study which compared antibiotic prophylaxis with endoscopic injection and observation in children with VUR, the incidence of recurrent fUTIs was significantly lower in girls receiving antibiotic prophylaxis vs. observation (19 vs. 57% over a median period of 2 years; *p* = 0.0002) (72). In boys, the numbers of recurrent fUTIs were low in both study groups, with no significant difference. The RIVUR trial and others have demonstrated that delayed treatment of UTIs increases the risk of renal scarring (71, 73, 74). Despite the data showing possible benefits of antibiotic treatment, it is important to consider that VUR often persists for years, meaning that antibiotic prophylaxis is often needed for a prolonged duration. In contradiction to the studies above, a 2019 Cochrane review reported that long-term antibiotic prophylaxis “makes little or no difference to the risk of repeat UTI causing a person to be unwell” (34). Other studies also suggest that prophylactic antibiotic therapy can often be discontinued without incurring significantly increased UTI rates (75). The extent to which patients adhere to their prescribed treatment may explain some of the variability between studies, with real-world compliance rates tending to be considerably lower than those in clinical trials. In 2007, Hensle et al. reported a compliance rate of only 17%, suggesting widespread exposure to the same risk of UTIs as children under observation only (76).

The risk of antibiotic resistance in children receiving prophylactic antibiotics is an important consideration when choosing between management options (34, 71, 77, 78). Another possible drawback of antibiotic prophylaxis is deleterious effects on the microbiome of the gut, which can have a significant impact on patients' overall health (77, 79–81). These aspects are now recognized to a much greater extent than they were 20 years ago.

A cost-effectiveness analysis of antibiotic therapy was performed using results from the RIVUR trial (82). This study showed that antibiotic prophylaxis has marginally higher costs than placebo, while significantly reducing the incidence of infection. A second cost-utility analysis reported that antibiotic prophylaxis is only cost-effective if administered to patients with grade IV VUR; costs per quality-adjusted life-year gained in patients with grade I–III VUR were deemed prohibitively high (83).

In the future, it may become possible to better select specific patients who would benefit from antibiotic prophylaxis (84). However, the viability of long-term antibiotic prophylaxis as a treatment option for all patients with VUR remains questionable.

### Endoscopic Injection

Clinical data from numerous studies have confirmed long-term safety and efficacy of endoscopic injection. In a meta-analysis published in 2016, the overall resolution rate ranged between 71 and 83%, depending on the injection technique. Studies with long-term follow-up [3–22 years, mostly performed with NASHA/Dx (Deflux)] have similarly reported resolution rates ranging between 69 and 100% (28, 85–88). In addition to results in “uncomplicated” VUR, numerous studies have provided evidence that endoscopic injection is also effective in specific patient populations that may be deemed more difficult to treat (historically not considered for endoscopic therapy). These include high-grade VUR, duplicated systems, adult women and kidney transplant patients (85, 86, 89–104). Resolution rates may be reduced in these groups of patients: for example, Läckgren et al. reported a positive response rate of 63% in patients with duplicated ureters, compared with 68% in the broader population of VUR patients (104, 105). However, the success rates are high enough for endoscopic injection to remain viable in these groups of patients.

A range of factors have been shown to influence the resolution rate with endoscopic injection. Statistically significant effects on outcomes have been reported with VUR grade, injection technique, physician experience, patient age, and the extent of renal scarring at time of treatment (35, 106–108). In addition, high UDR values have been associated with reduced likelihood of VUR resolution following endoscopic injection (109). Baydilli et al. recently studied associations between a range of clinical parameters and the outcome of endoscopic therapy with NASHA/Dx (110). The factors associated with greatest increase in the likelihood of failure of NASHA/Dx to resolve VUR were: onset of reflux during the early filling phase of the voiding cycle, UDR value above 0.24, and a delay in upper urinary tract drainage after voiding. Presence of renal scarring, presence of BBD, history of fUTI and high-grade VUR were also associated with significantly increased risk of treatment failure.

There is little evidence of major differences in VUR resolution rates between injectable agents in current use (34). This appears contingent upon formation of a long-lasting bolus following injection; experience with bovine collagen indicated lower efficacy than with other injectable agents (111–113). This was attributable to degradation of collagen post-injection, and collagen is not currently used for endoscopic treatment of VUR. The choice of injectable agent may have a more significant impact on the safety of endoscopic injection. Early investigations of endoscopic injection were performed using polytetrafluoroethylene (PTFE) and polydimethylsiloxane (silicone). Safety concerns with these products include granuloma formation (a foreign-body reaction), migration from the injection site, and, because of their lack of biodegradability, permanent accumulation within the body (113–115). These considerations led to PTFE and silicone falling out of common use in patients with VUR.

Recently developed injectable agents include polyacrylate-polyalcohol copolymer, polyacrylamide hydrogel, and small-size (80–120 μm) dextranomer/hyaluronic acid copolymer (116). Like PTFE and silicone, polyacrylate-polyalcohol copolymer and polyacrylamide hydrogel are non-biodegradable, meaning they can remain within the body permanently. They both have a favorable histopathologic profile, but foreign-body reactions are possible (117–120). Polyacrylate-polyalcohol copolymer has been associated with risks of periureteral fibrosis (potentially complicating subsequent ureteral reimplantation) and obstruction of the vesicoureteral junction (116, 121). Comparative studies suggest that polyacrylate-polyalcohol copolymer, polyacrylamide hydrogel and small-size dextranomer/hyaluronic acid copolymer are at least as effective as NASHA/Dx in resolving VUR (106, 121–124). Only one of these studies was a prospective, randomized trial; the results showed comparable efficacy with NASHA/Dx and polyacrylate-polyalcohol copolymer (121). The only other prospective study (non-randomized) also reported similarity between the two agents being compared (NASHA/Dx and polyacrylamide hydrogel) (123). The remaining comparisons of recently developed materials vs. NASHA/Dx were retrospective, limiting the robustness of the results. Small-size dextranomer/hyaluronic acid copolymer (brand names Urodex, Vurdex and Dexell) differs from NASHA/Dx (brand name Deflux) not only in the size of the dextranomer microspheres, but also in the characteristics of the hyaluronic acid, potentially affecting the safety profile, physical properties and ease/controllability of the injection procedure. Importantly, these differences mean that clinical results obtained with NASHA/Dx are not directly applicable to small-size dextranomer/hyaluronic acid copolymer. Long-term efficacy and safety data (>5 years) are yet to be published with any of the recently developed agents. We advocate NASHA/Dx because of its long-term safety (documented follow-up to 25 years), robust published evidence of efficacy and international regulatory approval (in the USA, it is the only FDA-approved material for endoscopic treatment of VUR).

A limited number of studies have assessed the pharmacoeconomics of endoscopic treatment of VUR. Early data published by Kobelt et al. in 2003 showed that, in the USA, endoscopic treatment with NASHA/Dx could reduce the cost of VUR management without reducing the clinical success rate (125). Another US study, published 3 years later, similarly reported that NASHA/Dx could be more cost-effective than ureteral reimplantation in patients with unilateral grade III VUR, although not in patients with bilateral grade III VUR or grade IV–V VUR, in whom larger volumes of NASHA/Dx are needed (126). In 2008, total reimbursement costs in the USA were found to be lower with outpatient ureteral reimplantation than with endoscopic injection for VUR (127). However, the cost difference was only ~10%, and total reimbursement for ureteral reimplantation was increased if a proportion of these patients require hospital admission. In 2016, results from patients treated in two European centers were analyzed to compare endoscopic treatment of VUR using NASHA/Dx with two methods of ureteral reimplantation (open Cohen and laparoscopic Lich-Gregoir) (128). Intra-operative costs were highest with endoscopic injection, but the total cost (intra-operative plus post-operative hospitalization costs) was highest with the Cohen procedure (€8201), and similar with endoscopic treatment and laparoscopic reimplantation (€3283 and €3211, respectively). Observations regarding lower product costs with polyacrylamide hydrogel and small-size dextranomer/hyaluronic acid copolymer vs. NASHA/Dx have been made in some publications (88, 123, 129). Simple comparisons of product costs do not provide a complete pharmacoeconomic picture: formal studies that include the total long-term costs of patient management (influenced by long-term safety and efficacy of the treatments concerned) are needed for true pharmacoeconomic comparisons.

### Ureteral Reimplantation

Ureteral reimplantation is associated with high resolution rates (>90%) in grade ≤ IV VUR. It is considered an invasive procedure that requires hospital admission and time for recovery (130–134). There is a small risk of post-operative complications; these occur in ~5–9% of children undergoing open surgery (134, 135).

Laparoscopic and robotic methods have the potential to reduce the invasiveness of ureteral reimplantation, and these methods are gaining popularity (136). A multicenter, retrospective analysis of laparoscopic ureteral reimplantation conducted in patients with grade II–IV VUR reported a success rate of 96% (137). A 2016 review of laparoscopic ureteral reimplantation also reported a median success rate of 96%, with a complication rate of 7% (138). Success rates similar to those with open surgery (>90%) have been reported with robot-assisted laparoscopic ureteral reimplantation, although there is evidence that the success rate with this method may be lower (around 80%) when the procedure is performed bilaterally (139–141). Urinary retention has been reported as a complication among patients undergoing robot-assisted ureteral reimplantation, and the overall complication rate appears higher in patients undergoing bilateral procedures (140, 142). The cost-effectiveness of robot-assisted ureteral reimplantation has been questioned due to higher costs and higher complication rates compared with open surgery (135). The learning curve for robotic surgery can be substantial, and is best done at centers with high patient numbers (143–145). In addition, the costs associated with procuring robotic equipment may limit the availability of this approach. Treatment outcomes are likely to improve as techniques are developed further, but current data indicate that open surgery may still be preferable.

### Cochrane Review

A recently published Cochrane review evaluated benefits and harms of all the available interventions for VUR. Thirty-four randomized studies met the inclusion criteria (34). Antibiotic prophylaxis was reported to have little effect on the risk of UTI and to increase the likelihood of antibiotic resistance. The benefits with endoscopic injection or ureteral reimplantation vs. antibiotic treatment were deemed unclear due to insufficiencies in study design.

## Management Recommendations

The main aim of VUR management recommendations is to ensure that each patient receives the most appropriate intervention for their individual needs. There are variations between countries in the approach to VUR management and in the specialty of the healthcare provider who first sees the patient. International variability is also encountered in the licensed indications for devices including the injectable agents used in endoscopic treatment.

### Developments Over the Last 20 Years

The 1997 AUA guidelines recommended antibiotic prophylaxis as first-line treatment, with surgery (ureteral reimplantation) as second-line treatment for persistent cases or as first-line intervention in severe VUR (particularly in older children) (146). Endoscopic injection was not recommended for routine use at that time, and concerns we now have regarding antibiotic prophylaxis were less well-understood. In 2002, positive results obtained with endoscopic injection of NASHA/Dx led to the proposal of an updated treatment algorithm (147). For most patients, 1 year of antibiotic prophylaxis was recommended in the first instance. For those in whom VUR persisted to the end of the year, endoscopic injection was proposed. Ureteral reimplantation was considered appropriate for patients not responding to endoscopic treatment, and it was also recommended as first-line intervention in high-risk groups (children aged >1 year with grade V reflux, and those aged >5 years with grade bilateral III–IV reflux) (147).

Over the last 20 years, there has been a shift toward more conservative management of VUR. More emphasis is now placed on an individualized, risk-based approach, with less reliance on long-term antibiotic prophylaxis, reduced use of VCUG and a decline in surgical intervention (45). Also, patients with concurrent VUR and BBD are understood to have an increased risk of UTI vs. patients with VUR only, meaning that treatments for both conditions may be needed (22, 25). It remains unclear whether BBD should always be treated before VUR.

### Current Guidelines

The European Association of Urology (EAU) guidelines on the management of VUR are 8 years old (69). They state prominently that “there is no consensus on the optimal management of VUR or on its diagnostic procedures, treatment options, or most effective timing of treatment.” VUR guidelines from the American Urological Association (AUA) were updated more recently, in 2017, but these too include a comment that “the data were not sufficient to permit development of strict ‘standards of care' in many instances” (68). This has undoubtedly contributed to the current variability in VUR management.

The EAU and AUA guidelines recommend VCUG in infants with prenatally diagnosed hydronephrosis, and siblings and offspring of VUR patients (68, 69). EAU guidelines also recommend VCUG examination in children with an fUTI or lower urinary tract dysfunction (69). For infants diagnosed in the first year of life, antibiotic prophylaxis is recommended as first-line treatment, with ureteral reimplantation or endoscopic treatment for those with breakthrough infections (68, 69). Antibiotic prophylaxis is also recommended in the EAU guidelines as initial treatment for children aged 1–5 years with grade III–V VUR, although ureteral reimplantation should be considered as an alternative in those with high-grade VUR (69). For children with lower urinary tract dysfunction (LUTS) as well as VUR, the EAU recommend that initial management should be focused on LUTS. Endoscopic treatment is recommended principally as an option for children with low grades of VUR (up to grade III) and, for high-risk patients with renal impairment, an “aggressive, multidisciplinary approach” is recommended (69). In the AUA guidelines, for patients aged >1 year and no BBD, antibiotic therapy is suggested as an option, while endoscopic injection or ureteral reimplantation are recommended for patients with recurrent UTIs or new renal abnormalities. For patients aged >1 year with concurrent VUR and BBD, the AUA guidelines recommend antibiotic therapy with BBD treatment (68). The AUA guidelines describe lower success rates with endoscopic injection vs. ureteral reimplantation, but definite recommendations on how to choose between these options are lacking (68).

Guidelines on managing patients with UTIs also include recommendations relating to VUR. The American Academy of Pediatrics (AAP) guidelines of 2011 were influential in reducing the use of VCUG. Before 2011, patients between 2 and 24 months of age with an fUTI routinely underwent VCUG assessment. In contrast, the 2011 guidelines recommended renal and bladder ultrasound assessment for patients with their first fUTI, and no VCUG among those without ultrasound-detectable abnormalities (148). This approach was reaffirmed by the AAP in 2016 (149). The UK National Institute for Health and Care Excellence (NICE) similarly recommend VCUG only in selected children with UTIs: those aged <6 months with atypical or recurrent UTIs (150). These UK guidelines recommend surgical treatment of VUR (either endoscopic injection or ureteral reimplantation) only for VUR patients with “symptomatic breakthrough UTIs despite medical management and/or increased renal parenchymal defects.”

### Current Opinion of the Authors

**Figure 2:**
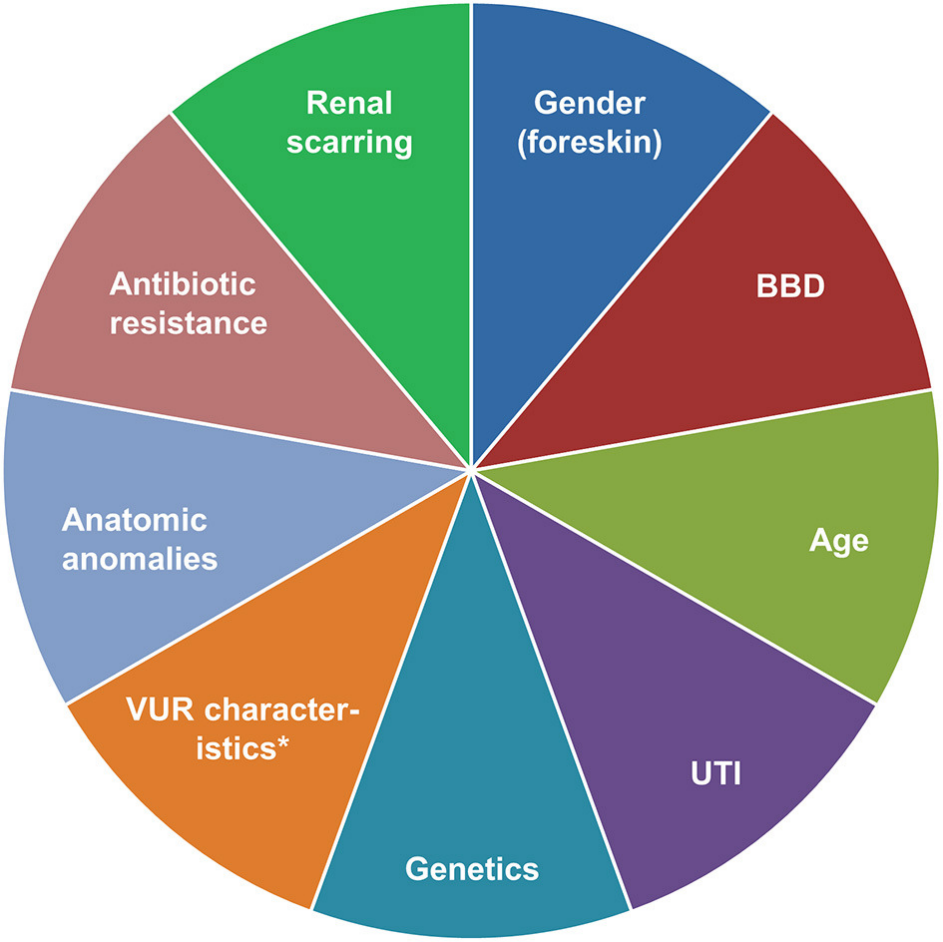
Factors to consider in a risk-based approach to the management of VUR. *Grade, bladder volume at onset of reflux, ureteral diameter ratio. BBD, bladder/bowel dysfunction; UTI, urinary tract infection; VUR, vesicoureteral reflux.

In all patients with VUR, there is a need to balance risks, benefits and costs of treatment vs. risks (particularly to the kidneys) of not treating the condition (Figure 2) (14, 33, 35, 151). We believe that first-line endoscopic injection is preferable for many VUR patients requiring intervention. Ureteral reimplantation is usually performed in patients not responding to endoscopic injection, those with primary obstructive refluxing megaureter, and those with grade V VUR and concomitant narrowing of the vesicoureteral junction. For patients with VUR and bladder or bowel dysfunction (BBD), we recommend treating BBD as early as possible (before VUR intervention). However, in cases with recurrent breakthrough UTIs, endoscopic treatment or ureteral reimplantation should not be delayed and BBD therapy can still be undertaken as needed. We no longer support the routine use of long-term antibiotic prophylaxis for VUR. Long-term monitoring of patients with VCUG assessments after endoscopic treatment or ureteral reimplantation appears unnecessary, due to the high cure rates with both treatment options. Follow-up VCUGs are mainly triggered by the occurrence of symptomatic UTIs. Every decision needs to be taken with due consideration of the individual patient's history and current health status, risk of recurrent UTI, as well as the wishes of the patient and/or their parents.

## Conclusions

Over the last 20 years, our understanding of VUR has increased considerably. A proportion of children with renal scarring after UTI (particularly those with grade III–V VUR and recurrent fUTI) are at risk of renal deterioration. Improved knowledge of how to identify such patients has led to an individualized, risk-based approach to the management of VUR and an overall shift to more conservative management of VUR. Surgical methods of ureteral reimplantation have progressed but our opinion is that endoscopic injection is frequently preferable, based on evidence from the last two decades confirming the long-term tolerability and durability of this procedure. Although a number of materials have been explored as injectable agents during the last 20 years, NASHA/Dx is widely considered the preferred choice with the strongest long-term efficacy and safety data. Key knowledge gaps include the need for better identification of children at risk of recurrent UTIs and future renal scarring, robust evidence from randomized controlled trials, further evaluation of the side effects of chronic antibiotic exposure, and an improved VUR grading system. These gaps will need to be addressed in the coming years to ensure that individual patients' needs are fulfilled to the greatest possible extent.

## Author Contributions

All authors reviewed the literature and collaborated in writing and editing the manuscript.

## Conflict of Interest

GL: speaker at teaching courses for Ferring AB, Sweden; Medical adviser and speaker at instructional courses for Palette AB, Sweden. CC: consultant for Palette Life Sciences, Santa Barbara, CA USA. AK: speaker, consultant, and course director of instructional seminars for Palette Life Sciences. The remaining author declares that the research was conducted in the absence of any commercial or financial relationships that could be construed as a potential conflict of interest.
